# Protocol for *ADDITION-PRO*: a longitudinal cohort study of the cardiovascular experience of individuals at high risk for diabetes recruited from Danish primary care

**DOI:** 10.1186/1471-2458-12-1078

**Published:** 2012-12-14

**Authors:** Nanna B Johansen, Anne-Louise S Hansen, Troels M Jensen, Annelotte Philipsen, Signe S Rasmussen, Marit E Jørgensen, Rebecca K Simmons, Torsten Lauritzen, Annelli Sandbæk, Daniel R Witte

**Affiliations:** 1 Steno Diabetes Center A/S, Gentofte, Denmark; 2 Department of Endocrinology-Gastroenterology, Bispebjerg Hospital, Copenhagen University Hospital, Copenhagen, Denmark; 3 MRC Epidemiology Unit, Cambridge, UK; 4 Department of Public Health, Section of General Practice, Faculty of Health Sciences, Aarhus University, Aarhus, Denmark; 5 Centre de Recherche Public de la Santé, Luxembourg

**Keywords:** Diabetes, Cardiovascular disease, Primary care, Complications, Microvascular, Impaired fasting glucose, Impaired glucose intolerance, Aortic stiffness, Physical activity, Body composition

## Abstract

**Background:**

Screening programmes for type 2 diabetes inevitably find more individuals at high risk for diabetes than people with undiagnosed prevalent disease. While well established guidelines for the treatment of diabetes exist, less is known about treatment or prevention strategies for individuals found at high risk following screening. In order to make better use of the opportunities for primary prevention of diabetes and its complications among this high risk group, it is important to quantify diabetes progression rates and to examine the development of early markers of cardiovascular disease and microvascular diabetic complications. We also require a better understanding of the mechanisms that underlie and drive early changes in cardiometabolic physiology. The *ADDITION-PRO* study was designed to address these issues among individuals at different levels of diabetes risk recruited from Danish primary care.

**Methods/Design:**

*ADDITION-PRO* is a population-based, longitudinal cohort study of individuals at high risk for diabetes. 16,136 eligible individuals were identified at high risk following participation in a stepwise screening programme in Danish general practice between 2001 and 2006. All individuals with impaired glucose regulation at screening, those who developed diabetes following screening, and a random sub-sample of those at lower levels of diabetes risk were invited to attend a follow-up health assessment in 2009–2011 (n = 4,188), of whom 2,082 (50%) attended. The health assessment included detailed measurement of anthropometry, body composition, biochemistry, physical activity and cardiovascular risk factors including aortic stiffness and central blood pressure. All *ADDITION-PRO* participants are being followed for incident cardiovascular disease and death.

**Discussion:**

The *ADDITION-PRO* study is designed to increase understanding of cardiovascular risk and its underlying mechanisms among individuals at high risk of diabetes. Key features of this study include (i) a carefully characterised cohort at different levels of diabetes risk; (ii) detailed measurement of cardiovascular and metabolic risk factors; (iii) objective measurement of physical activity behaviour; and (iv) long-term follow-up of hard clinical outcomes including mortality and cardiovascular disease. Results will inform policy recommendations concerning cardiovascular risk reduction and treatment among individuals at high risk for diabetes. The detailed phenotyping of this cohort will also allow a number of research questions concerning early changes in cardiometabolic physiology to be addressed.

## Background

The increasing global prevalence of diabetes has led several countries to propose or introduce screening programmes for diabetes in the past decade [1,2]. These screening programmes, which generally combine a structured assessment of risk factors for diabetes with a measure of glycaemia, will inevitably find more individuals at high risk for diabetes than those with undiagnosed prevalent disease. The *ADDITION-Europe* study [3] included a stepwise screening programme for diabetes in general practice in Denmark, the UK and the Netherlands, and identified more individuals with impaired fasting glycaemia (IFG) or impaired glucose tolerance (IGT) than those with screen-detected diabetes [4,5]. The screening programme also identified large numbers with normal glucose levels despite having one or more risk factors for diabetes or cardiovascular disease (CVD) [4]. While established guidelines for treating individuals with diagnosed diabetes are available [6], it remains unclear which treatment or prevention strategies should be introduced among those found to be at high risk of diabetes following screening. Development of diabetes can be prevented by very intensive lifestyle intervention among motivated individuals with IGT [7] but no trials of diabetes prevention have been performed in groups at lower absolute risk. Furthermore, there are no large-scale studies of the association between diabetes risk, dysglycaemia, and the development of the initial stages of diabetic micro- and macrovascular complications.

In order to make better use of the opportunities for primary prevention of diabetes and its complications afforded by the identification of large groups of at-risk individuals, there are a number of research questions that require investigation. It is necessary to accurately assess progression to diabetes in groups at different levels of risk and to examine the development of early markers of CVD and diabetic microvascular complications. A better understanding of the mechanisms that underlie and drive early changes in cardiometabolic physiology is also required.

To investigate these issues, we invited individuals at different levels of diabetes risk identified from the stepwise screening programme of the Danish arm of the *ADDITION-Europe* study [3] to take part in a longitudinal cohort study (*ADDITION-PRO*). The overall aim of *ADDITION-PRO* is to increase understanding of CVD risk and its underlying mechanisms among individuals at different levels of diabetes risk. We will quantify the incidence of diabetes and CVD in high risk individuals, and examine diabetes and CVD progression using detailed measures of anthropometry, body composition, biochemistry, aortic stiffness and lifestyle behaviours. Results will inform policy recommendations concerning CVD risk reduction and treatment among individuals at high risk for diabetes.

Specific objectives of the *ADDITION-PRO* study include:

■ To quantify progression rates from groups at different levels of diabetes risk to IFG, IGT and diabetes, and to examine the determinants of glycaemic state transition

■ To establish whether initial levels of glycaemia and subsequent transitions from one glycaemic state to another affect the initial stages of micro- and macrovascular complications, and the risk of incident CVD and mortality

■ To examine the association between:

– objectively measured physical activity and markers of glucose homeostasis

– anthropometric and body fat distribution measures, markers of glucose homeostasis and the initial stages of micro- and macrovascular complications

– deterioration in glucose metabolism, long-term glycaemia and the development of initial stages of micro- and macrovascular complications

## Methods/Design

The *ADDITION-PRO* study is nested within the Danish arm (*ADDITION-DK*) of the *ADDITION-Europe* study [3,8]. *ADDITION-DK* consists of two phases: a population-based stepwise screening programme for type 2 diabetes and a randomised controlled trial of early intensive treatment among those found to have diabetes. A subset of individuals identified at high risk for diabetes following the screening phase of the study were invited to take part in the longitudinal *ADDITION-PRO* cohort study. Ethical approval was obtained from the scientific ethics committee in the Central Denmark Region (no: 20000183). Participants gave written informed consent to take part in the study and for linkage of their data with national registers for the purposes of the *ADDITION-PRO* study.

### *ADDITION-DK* stepwise screening programme

Full details of the screening programme are reported elsewhere [9,10]. In brief, a population-based, stepwise high-risk screening programme was performed in 190 general practices (GP) in five counties covering urban, suburban and rural areas of Denmark (Copenhagen, Aarhus, Ringkøbing, Ribe and South Jutland) from 2001 to 2006. Individuals eligible for invitation to screening were people registered with one of the participating general practices, aged 40 to 69 years, and not known to have diabetes. Exclusion criteria were assessed by the general practitioners and included pregnancy or lactation, being housebound, having a psychological or psychiatric problem likely to invalidate informed consent, or having an illness with a likely prognosis for life expectancy of less than one year. Eligible individuals received a slightly modified version of the Danish diabetes risk questionnaire by post or were asked to complete the questionnaire opportunistically while visiting their GP [11,12]. Recipients were asked to indicate known risk factors for diabetes including age, sex, BMI, known hypertension, family history of type 2 diabetes, gestational diabetes and leisure time physical activity. Those with a risk score ≥ 5 points were invited to continue in the stepwise screening programme, which included random blood glucose and HbA_1c_ testing, a fasting blood glucose test, and an oral glucose tolerance test (OGTT). World Health Organisation criteria were used to diagnose diabetes [13].

**Figure 1:**
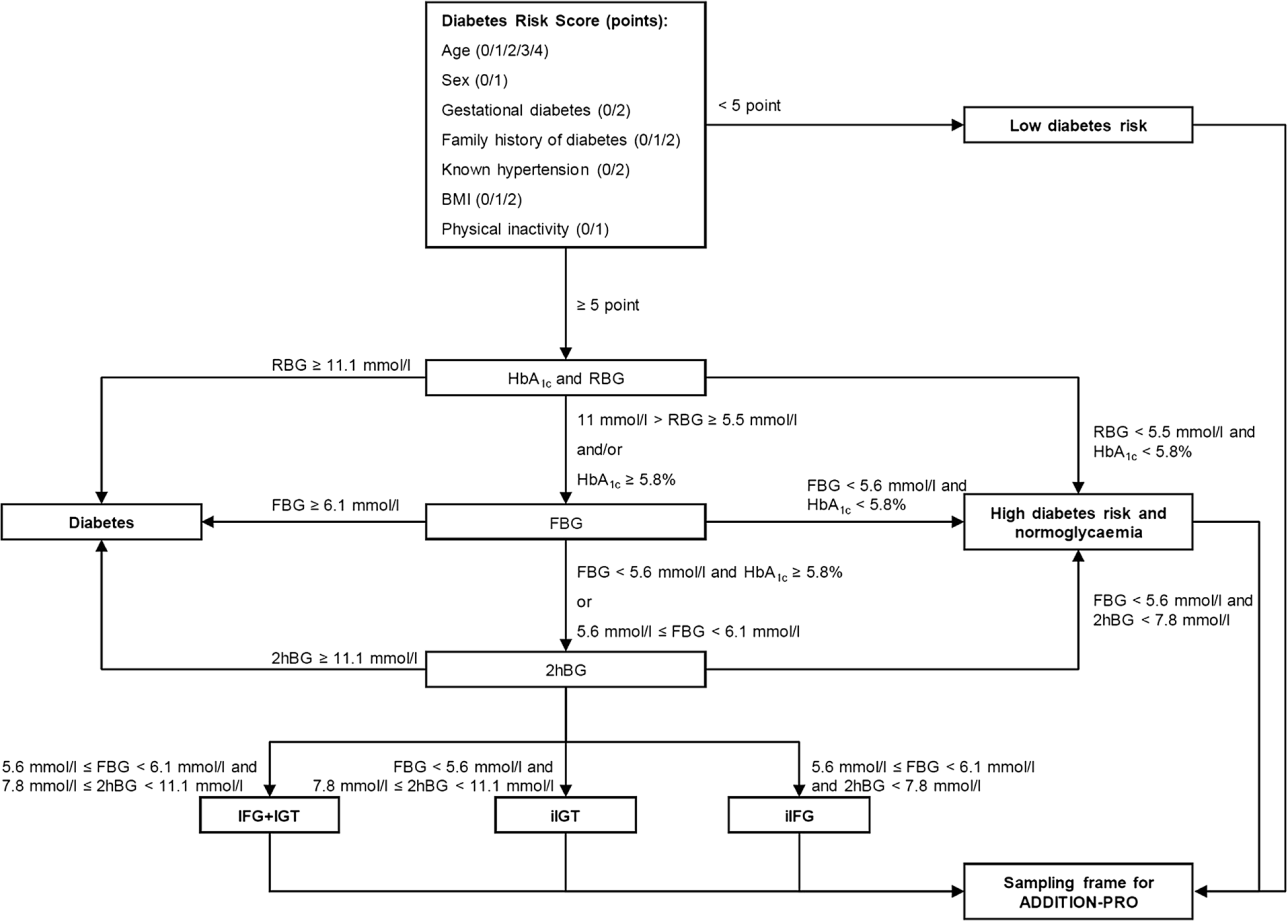
***ADDITION-DK *screening programme.**

In *ADDITION-DK*, 163,189 individuals aged 40–69 years were mailed the risk score questionnaire or filled it in opportunistically at their GP. 26,491 had a diabetes risk score ≥ 5 points and attended for a random blood glucose and/or HbA_1c_ measurement. Participants who completed the screening programme and who were not found to have diabetes (n = 22,200) were classified into groups of increasing diabetes risk: (i) high diabetes risk (≥5 points on diabetes risk score) with normoglycaemia (n = 19,968); (ii) isolated IFG (n = 918); (iii) isolated IGT (n = 769); and (iv) IFG and IGT (n = 545). In order to establish a group at low risk of diabetes, between 2001 and 2002, a sub-group of participants in Aarhus and Copenhagen county from both the postal and opportunistic screening programmes (n = 32,894) were asked to return their risk questionnaire regardless of their score. 13,288 individuals with a low diabetes risk (<5 points on diabetes risk score) were identified [12]. These stratified risk groups constitute the sampling frame for the *ADDITION-PRO* study (Figure 1).

### *ADDITION-PRO* study

**Figure 2:**
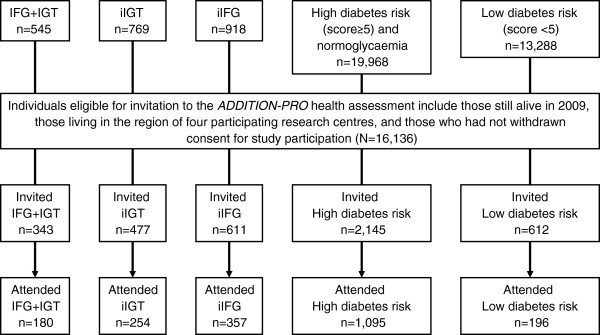
**Design and participant flow in the *ADDITION-PRO *study.**

In 2009–2011, a follow-up health examination of a subset of the stratified high risk population was performed at four out of the five original *ADDITION-DK* study centres to establish a longitudinal cohort. Individuals who were eligible to be invited included those (i) who were still alive, (ii) who lived in the regions of the four research centres (Steno Diabetes Center, Aarhus University Hospital, Holstebro Hospital, and Hospital of South West Jutland, Esbjerg), and (iii) who had not withdrawn consent to study participation. Of these 16,136 people, all individuals with impaired glucose regulation at the time of screening and those who were diagnosed with diabetes during the follow-up period before the time of invitation were invited (n = 1,483) as well as a 19% random sample of individuals from the low and high risk groups (n = 2,705). In total, 2,082/4,188 (50%) people agreed to participate and attended the *ADDITION-PRO* health assessment (Figure 2). Compared to attenders, non-attenders were more likely to be women and to have a normal weight, and were less likely to have a family history of diabetes (Table 1). Participation did not differ in terms of age, hypertension, history of gestational diabetes, or physical activity.

**Table 1:** **Difference in characteristics at screening between attenders and non-attenders to the *ADDITION-PRO *study**

**Characteristics at screening**		**Attenders n = 2,082**	**Non-attenders n = 2,106**	**p-value**^**1**^
Men (%)		53.4	42.8	< 0.001
Median age (years)		58.5	59.3	0.551
Gestational diabetes (% of women)		2.2	1.4	0.185
Family history of diabetes (%)	No relatives	67.7	73.8	< 0.001
	1 relative	24.3	19.6	
	2 relatives	6.1	5.0	
Hypertension^2^ (%)		36.7	36.5	0.838
Weight (%)	Normal weight	36.0	40.2	0.006
	Overweight	45.0	40.3	
	Obese	17.8	18.1	
Physically inactive^3^ (%)		78.1	78.1	0.856

^1^ p-value from t-tests or chi-squared test for the difference between attenders and non-attenders.

^2^ Hypertension defined as “has a doctor ever told you that you have high blood pressure” in the risk score questionnaire [12].

^3^ Classified as inactive from four questions pertaining to leisure time physical activity in the risk score questionnaire [14].

#### ADDITION-PRO health assessment

Health assessments were performed by trained staff following standard operating procedures. See Table 2 for a full list of measures taken during the screening programme and at the *ADDITION-PRO* health assessment.

**Table 2:** **Measures used at the screening phase (2001–06) and at the health assessment (2009–11) in the *ADDITION-PRO *study**

	**Screening phase: low risk**^**1 **^**participants**	**Screening phase: high risk**^**2 **^**participants**	***ADDITION-PRO *health assessment**
Danish risk score questionnaire	X	X	X
*Socio-demographic variables*			
Age	X	X	X
Sex	X	X	X
*Biochemistry*			
Random capillary blood glucose		X	
Fasting capillary blood glucose^3^		X	
Venous glycated haemoglobin (HbA_1c_)		X	X
75 g oral glucose tolerance test^3,4^		X	X
Insulin			X
Total cholesterol		X	X
LDL-cholesterol		X	X
HDL-cholesterol		X	X
Triglycerides		X	X
Plasma and urine creatinine^3^		X	X
Urine albumin^3^		X	X
DNA^3^		X	X
Alanine transaminase			X
Alkaline phosphatase			X
Biobank: plasma, serum, spot urine^3^		X	X
Whole saliva^5^			X
*Clinical measures*			
Electrocardiogram			X
Heart rate			X
Brachial blood pressure		X	X
Central blood pressure			X
Aortic pulse wave velocity			X
Advanced glycation end-products			X
*Anthropometric variables*			
Height		X	X
Weight		X	X
Waist circumference		X	X
Hip circumference		X	X
Body fat percentage			X
Abdominal fat distribution by ultrasound^6^			X
Hepatic fat content by ultrasound^6^			X
*Physical activity*			
Combined accelerometer and heart rate monitor (ActiHeart)			X
Recent physical activity questionnaire (RPAQ)			X
Sleep questionnaire^7^			X
*Questionnaire measures*			
Ethnicity / nationality		X	
Education		X	
Occupation		X	X
Personal medical history		X	X
Family history of diabetes	X	X	X
Family history of CVD		X	X
Current medication			X
Recent hospital admissions			X
Gestational diabetes (women only)	X	X	X
Lifestyle behaviours (smoking, alcohol consumption, physical activity)		X	X
Current weight, birth weight, weight loss/gain, perceived body image			X
EuroQol 5-D (health utility)			X
SF-36 (functional status)			X
*Registry information*			X
Cardiovascular disease	X	X	X
Type 2 diabetes	X	X	X
Medication use	X	X	X
Health service use	X	X	X
Mortality			X

^1^ Low diabetes risk <5 points on the diabetes risk score.

^2^ High diabetes risk ≥5 points on the diabetes risk score.

^3^ At screening, only for individuals with elevated RBG/FBG levels who progressed through to the later stages of the screening programme.

^4^ At *ADDITION-PRO*, only for individuals without incident diabetes since screening.

^5^ Only collected at the Steno Diabetes Center.

^6^ Only collected at the Steno Diabetes Center and in Aarhus.

^7^ Collected at the Steno Diabetes Center, Esbjerg and Aarhus from July 2010.

#### Clinical measures

A ten second 12-lead electrocardiogram (ECG) was taken with the participant in a supine position. Brachial blood pressure and heart rate was measured three times after a 10 minute rest (Omron M6 comfort, Omron Healthcare, Milton Keynes, UK) with the participant in a sitting position. The average of the three measurements for each parameter constitutes the values for brachial systolic and diastolic blood pressure and heart rate.

Central haemodynamics (aortic pulse wave velocity (aPWV) and central blood pressure) were assessed by applanation tonometry using a SphygmoCor device (version 8, Atcor Medical, West Ryde, NSW, Australia) and a high fidelity tonometer. With the participant in supine position after 10 minutes of rest, the velocity of the pulse wave was assessed between the right carotid and femoral artery. This is a validated method of assessing aortic stiffness [15], an assessment of subclinical organ damage. The tonometer was used to capture wave forms at the carotid and subsequently at the femoral artery simultaneously with an ECG recording using the intersecting tangent [16]. The transit time was based on the mean of ten pulse waves. The distance from the suprasternal notch to the carotid artery was measured with a tape measure and from the suprasternal notch to the femoral artery with an anthropometer (Seca, Medical Scales and Measuring Systems, Hamburg, Germany). The anthropometer was used to avoid overestimation of the distance and consequently the velocity in obese individuals. The path length was determined by subtracting the carotid-sternal notch distance from the femoral-sternal notch distance. In each participant, aPWV was measured twice. If the difference in aPWV between the two measurements was larger than 0.5 m/s, a third measurement was taken. The average of the two closest measurements in each participant constitutes the value of aPWV.

Central blood pressure, augmented pressure and augmentation index were calculated from the peripheral pressure waveforms recorded at the radial artery with the participant in the supine position. The radial waveforms were calibrated by the supine brachial systolic and diastolic blood pressure based on a built-in generalised transfer function. Supine brachial blood pressure was measured after a 10-minutes rest with an automated oscillometric blood pressure recorder (Omron M6 comfort). From the central waveforms, central systolic and diastolic blood pressure, pulse pressure, augmented pressure and augmentation index were estimated. At least two measurements were taken, and the average of each pressure index constitutes the values of central systolic blood pressure, diastolic blood pressure, pulse pressure, augmentation index and augmented pressure.

Advanced glycation end-products (AGE) were measured by skin autofluorescence using an AGE-reader (Diagnoptics Technologies B.V, Groningen, The Netherlands). The measurement is based on illumination of a ~ 4 cm^2^ area of skin on the volar side of the forearm with light (wavelength of 300–420 nm), which excites fluorescent moieties in the skin. Autoflourescence, the fluorescent light subsequently emitted from the skin (wavelengths of 420–600 nm), is measured by an integrated spectrometer and expressed as the ratio between the intensity of the emitted fluorescent light and the excitation light [17]. AGE measurements were taken on the right forearm preceded by the removal of dead skin cells with alcohol preparation pads. One measurement session consisted of three AGE measures, and in each participant three sessions were completed. The average of the three sessions is regarded as the AGE value.

#### Anthropometric measures

Height was measured to the nearest millimetre using a fixed rigid stadiometer (Seca, Medical Scales and Measuring Systems, Hamburg, Germany). Weight was measured in light indoor clothing without shoes to the nearest 0.1 kg with a Tanita Body Composition Analyser (Tokyo, Japan). Clothes were estimated to weigh 0.5 kg and this weight was deducted from the total weight. Waist and hip circumference were measured with the participant in a standing position using a D-loop tape. Waist circum-ference was measured at the mid-point between the lower costal margin and the level of the anterior superior iliac crest to the nearest millimetre and hip circumference was measured at the widest level of the hips. Waist and hip measurements were completed twice. If the difference between two consecutive measurements was more than 3 cm, a third measurement was taken. The mean of the two closest measurements constitutes the values of waist and hip circumference. Body fat percentage was assessed by bio-electrical impedance using the TANITA analyser. Measurements were registered with the participant standing barefoot on the weighing platform. Participants with pacemakers or other internal medical devices were measured only using SECA scales.

#### Body fat distribution

Abdominal fat distribution and hepatic fat content were assessed by ultrasonography (Logiq9 ultrasound machine, GE Healthcare, Waukesha, WI, USA) at two study centres (Steno Diabetes Center and Aarhus) [18,19]. With the participant lying down, the transducer was placed on the abdomen where the xiphoid line crosses the waist circumference (described above). Measurements were performed at the end of a quiet expiration using minimal pressure on the transducer. Subcutaneous fat was recorded as the vertical distance from the skin to the linea alba with a 9 L (2.5-8.0 MHz) transducer in the transverse position. Visceral fat was recorded at the same location, with a 4 C (1.5-4.5 MHz) transducer placed longitudinally, as the vertical distance from the peritoneum to the front edge of the vertebra. In order to quantify hepatic fat content an image of the liver was captured intercostally with the participant in the supine position, with the right arm held above the head. A curved array 4 C transducer (3 MHz) was used. All ultrasound machine settings were standardised to allow subsequent analysis of image characteristics to quantify hepatic fat content.

#### Objectively measured physical activity

Physical activity was measured using a combined accelerometer and heart rate monitor (ActiHeart®, CamNTech Ltd., Cambridge, United Kingdom) [20]. The monitor was placed horizontally on the participants’ chest on two standard electrocardiogram electrodes (Maxensor, Alton, United Kingdom), one at the lower part of the sternum and the other one on the same horizontal level, on the left side, as laterally as possible. On the day of the health examination, a sub-maximal step test was performed to allow individual calibration of the heart rate to physical activity intensity [21]. The eight-minute step test was administered from the Actiheart software to indicate the cycles of stepping up and down a 20.5 cm step bench (Rucanor Europe B.V., Nieuwerkerk, The Netherlands). The stepping frequency ranged from 15 to 33 step cycles per minute over the duration of the test (8-minutes), followed by a two-minute recovery period (sitting). After the participant had completed the step test, the monitor was set up to record free-living physical activity, registering movement and heart rate every 60 seconds. Participants were asked to wear the monitor for seven days and nights and to maintain their usual physical activity pattern during the period. Alongside wearing the monitor, participants were asked to fill in a log to register any non-wear time and comments during the measuring period. Heart rate and accelerometry measures from the Actiheart monitor were downloaded using the manufacturer’s software (http://www.camntech.com). These measures were then cleaned and processed to reduce noise, outliers, and incomplete heart rate measures [22]. Physical activity measures were derived by combining minute-to-minute heart rate and accelerometry measures using a branched equation model [23].

#### Biochemical measures

Spot urine for analysis of albumin and creatinine was collected in plastic containers. Venous blood samples were drawn after an overnight fast (≥8 hours). Participants without known diabetes underwent a standard 75 g OGTT with blood samples drawn at 30 and 120 minutes. Plasma for analysis of glucose was prepared immediately upon collection in fluoride-heparin coated tubes. Samples were placed on ice before centrifugation at 3000 rpm for 10 minutes at 4°C. Plasma for analysis of creatinine, total cholesterol, HDL-cholesterol, and triglycerides was prepared upon collection in lithium-heparin coated tubes by incubating for 0.5-1.5 hours at room temperature with subsequent centrifugation at 3000 rpm for 10 minutes without cooling. Serum for analysis of insulin was prepared by incubating whole blood for 0.5-1.5 hours at room temperature with subsequent centrifugation for 10 minutes at 3000 rpm without cooling. Whole blood for analysis of HbA_1c_ and DNA was collected in EDTA coated tubes. Additionally, aliquots of plasma (0, 30, 120 min), serum (0, 30, 120) and spot urine were collected for the *ADDITION-PRO* biobank. Plasma for the biobank was collected in chilled EDTA coated tubes and centrifuged within 30 minutes at 3000 rpm for 10 minutes at 4°C. Biobank samples were subsequently stored at −80°C.

All biochemical measures were analysed at the Clinical Chemistry Department at the Steno Diabetes Center in Gentofte, Denmark. Serum insulin was measured by immunoassay (AutoDELFIA, Perkin Elmer, Massachusetts, United States). Glycated haemoglobin A_1c_ (HbA_1c_) was measured by HPLC (TOSOH G7, Tokyo, Japan). Between 2009 and 2010, plasma glucose, alanine transaminase, alkaline phosphatase, total cholesterol, HDL-cholesterol, triglycerides, plasma creatinine, urine creatinine and urine albumin were measured using the Hitachi 912 system (Roche Diagnostics, Mannheim, Germany). During 2010, the study laboratory gradually implemented the Vitros 5600 Integrated System (Ortho Clinical Diagnostics, Illkirch Cedex, France). There was modest agreement between the Hitachi 912 and Vitros 5600 instruments. Thus, all ‘Vitros’ values were converted to correspond to ‘Hitachi’ values, using regression equations from validation analyses performed by the study laboratory (Table 3).

**Table 3:** **Conversion of biochemical measures in the *ADDITION-PRO *study**

**Biochemical measure**	**Equation**
Glucose	x = (y + 0.2637) / 0.983
Alkaline phosphatase	x = (y-8.7108) / 1.0465
Alanine transaminase	x = (y + 0.7823) / 0.9761
Plasma creatinine	x = (y-2.7403) / 1.0147
HDL cholesterol	x = (y + 0.2141) / 1.1254
Triglycerides	x = (y + 0.0196) / 1.1017
Total cholesterol	x = (1.0303*y-0.2362)
Urine albumin	x = (0.8861*y-0.6412)
Urine creatinine	x = (y-111.72) / 1.0087

x = value measured by Hitachi 912 machine.

y = value measured by Vitros 5600 machine.

Albumin creatinine ratio was calculated using the formula: U-albumin mg/l × 8.84)/(U-creatinine(μmol/l)/1000). VLDL cholesterol (VLDL-C) was calculated using the formula: VLDL-C = triglycerides (mmol/l)/2.2). VLDL-C was calculated only for triglyceride values ≤ 5.05 mmol/l. LDL cholesterol was calculated using Friedewald’s equation (LDL-C = TC – VLDL-C – HDL-C mmol/l) [24]. LDL-cholesterol was calculated only for triglyceride values ≤ 4.55 mmol/l.

Collection of whole saliva was performed at one of the study centres (Steno Diabetes Centre). Participants were asked not to brush teeth on the day of the health assessment. Upon arrival, participants were instructed to chew on paraffin wax for approximately one minute and then to empty their mouth of saliva. The participants were then instructed to chew on the paraffin wax for a further three minutes whilst spitting into a collection container whenever needed. The collected saliva was divided into two cryotubes. One tube was stored immediately at −80°C. RNAlater (Ambion, Austin, TX) was added to the second tube in a 1:3 ratio (saliva:RNAlater) and then refrigerated for approximately 24 hours before being transferred to −80°C storage.

#### Questionnaires

A general health questionnaire was mailed to each participant in advance of their measurement visit. The questionnaire included sections on personal medical history, family history of diabetes / CVD, and lifestyle behaviours (smoking status, weekly alcohol consumption, and physical activity). There were questions on current weight, birth weight, weight loss / gain and perceived body image, as well as commuting, occupational and leisure time physical activity habits throughout the life course. The questionnaire included the EuroQol (EQ-5D) health utility measure [25] and the SF-36 functional status measure [26]. Socio-demographic questions included age, occupation, nationality, and ethnicity. Data on current medication and recent hospital admittance were also collected. Medication was coded by a supervised nurse according to the Danish formulary (http://medicin.dk). Female participants were asked for details on the number of pregnancies and live births they had experienced, and for the number and birth weight of their children. They reported if they had developed gestational diabetes.

Physical activity during the last four weeks prior to filling out the questionnaire was assessed using a modified Danish version of the validated “recent physical activity questionnaire” (RPAQ) [27]. From July 2010 onwards a detailed sleep questionnaire was completed alongside combined heart rate and accelerometry measurement. All questionnaires were checked for completeness before the participant finished their measurement visit. The clinical research forms, general health questionnaire and RPAQ were scanned using a Teleform reader and verified manually. Double data entry of the sleep questionnaire was undertaken by experienced research assistants. All data were checked for outliers and nonsense values and cleaned before being uploaded to the study database.

#### Registry data

Data will be drawn from the unique register system in Denmark. The Danish National Civil Registry assigns a personal Civil Registration Number to all citizens in Denmark. All Danish citizens have National Health Insurance and are entitled to free access to medical care from general practice and hospitals. Data from different sources can be combined using the civil registration number. We will access the following registries to collect information on incident CVD, incident diabetes, mortality, medication and health service usage: the National Patient Registry (covering admissions and outpatient contacts to the hospitals), the National Health Service Registry (contacts in general practice), the Medical Prescription Registry, the Diabetes Registry, and the Death Cause Registry (based on death certificates, 100% coverage).

#### Sample size

Based on prior experience in the use of Statistics Denmark linkage for event follow up, we expect to achieve a 90% completeness of follow up for CVD events and a 100% completeness of follow up for mortality. Table 4 shows the power calculations performed prior to the start of the clinical examinations. The expected number of events in the *ADDITION-PRO* population was based on incidence rates and hazard ratios reported by the Hoorn study [28]. Based on expected mortality and attrition due to loss to follow up, we aimed to examine 1,800 participants who had IFG/IGT at screening, over half of whom were expected to progress to diabetes during follow-up. Adding a random subset of 900 participants who had normoglycaemia at screening yielded ample power to detect differences in CVD risk between the normoglycaemic group and the stable IFG/IGT and incident diabetes group, respectively. We examined 77% of the target population. One of the original *ADDITION-DK* study centres did not participate in the *ADDITION-PRO* health assessment and there was a lower than expected participation rate (50% rather than the expected 75%) amongst the group of participants with IFG/IGT. While the difference in CVD incidence between the stable IFG/IGT group and the incident diabetes group is expected to be smaller, comparison of the continuous measures of atherosclerosis, renal function and AGE accumulation will be sufficiently powered to highlight even small differences between all study groups.

**Table 4:** **Power calculations for main outcomes in the *ADDITION-PRO *study**


	**Expected CVD events (n)**	**Expected incidence rate (per 1000 person years)**	**Power**
Difference in CVD incidence between:			
NGT and stable IFG/IGT	53	7.4	0.97
NGT and incident DM	100	13.8	0.99
Stable IFG/IGT and incident diabetes	132	18.3	0.59
	**Detectable differences**	**Expected mean (SD) in reference group (NGT)**	**Power**
Aortic stiffness (pulse wave velocity)	0.4 m/s	7.5 m/s (2.5)	0.94
Renal function (eGFR)	5 ml/min/1.73 m^2^	100 ml/min/1.73 m^2^ (20)	0.98
AGE – (skin autofluorescence)	0.2 AU	2.0 AU (0.5)	0.94

*AGE* = advanced glycation end-products; *CVD* = cardiovascular disease;

*eGFR* = estimated glomerular filtration rate; *IFG* = impaired fasting glucose;

*IGT* = impaired glucose tolerance; *NGT* = normal glucose tolerance.

## Discussion

The *ADDITION-PRO* study is designed to increase understanding of CVD risk and its underlying mechanisms among individuals at high risk of diabetes recruited from a stepwise screening programme in Danish primary care. Key features of this study include (i) a carefully characterised cohort at different levels of diabetes risk; (ii) detailed measurement of anthropometric, body composition, biochemical and cardiovascular risk factors; (iii) objective measurement of physical activity behaviour; (iv) examination of the initial stages of micro- and macrovascular complications; and (v) long-term follow-up of hard clinical outcomes including mortality and CVD. Results will inform policy recommendations concerning CVD risk reduction and treatment among individuals at high risk for diabetes. The detailed phenotyping of this cohort will also allow a number of research questions concerning early changes in cardiometabolic physiology to be addressed.

## Abbreviations

AGE: Advanced glycation end-products; aPWV: Aortic pulse wave velocity; BG: Blood glucose; CVD: Cardiovascular disease; ECG: Electrocardiogram; EQ-5D: EuroQol health utility measure; FPG: Fasting plasma glucose; GP: General practice; HbA_1c_: Glycated haemoglobin; IFG: Impaired fasting glycaemia; IGT: Impaired glucose tolerance; NGT: Normal glucose tolerance; OGTT: Oral glucose tolerance test; RPAQ: Recent physical activity questionnaire.

## Competing interests

NBJ, ASH, AP, TMJ, and MEJ are employed by Steno Diabetes Center A/S, which is a research and teaching hospital collaborating with the Danish National Health Service and owned by Novo Nordisk A/S. NBJ, ASH, TMJ, AP, MEJ, TL and DRW hold shares in Novo Nordisk A/S. All other authors declare that they have no competing interests.

## Authors’ contributions

DRW, MEJ, TL and AS are principal investigators for the *ADDITION-PRO* study. SSR and DRW initiated and designed the study. NBJ, DRW, ASH, TMJ and AP were the study coordinators. NBJ, ASH, AP and TMJ trained the clinic staff in measurement techniques. ASH was responsible for physical activity measurements, AP for ultrasound measures, TMJ for biochemical measures and NBJ for all other clinical measurements. NBJ, DRW, ASH, TMJ, AP and RKS drafted the manuscript. All authors read and approved the final manuscript. DRW is the paper guarantor.

## Pre-publication history

The pre-publication history for this paper can be accessed here:

http://www.biomedcentral.com/1471-2458/12/1078/prepub
